# Preterm Delivery Disrupts the Developmental Program of the Cerebellum

**DOI:** 10.1371/journal.pone.0023449

**Published:** 2011-08-17

**Authors:** Parthiv Haldipur, Upasna Bharti, Corinne Alberti, Chitra Sarkar, Geetika Gulati, Soumya Iyengar, Pierre Gressens, Shyamala Mani

**Affiliations:** 1 National Brain Research Centre, Manesar, Haryana, India; 2 Inserm, CIE 5; Assistance publique - Hôpitaux de Paris, Robert Debré Hospital, Unité d'épidémiologie clinique, Paris, France; 3 Université Paris 7, Faculté de Médecine Denis Diderot, Paris, France; 4 All India Institute of Medical Sciences, New Delhi, India; 5 Inserm, U676, Paris, France; 6 PremUP, Paris, France; Hôpital Robert Debré, France

## Abstract

A rapid growth in human cerebellar development occurs in the third trimester, which is impeded by preterm delivery. The goal of this study was to characterize the impact of preterm delivery on the developmental program of the human cerebellum. Still born infants, which meant that all development up to that age had taken place in-utero, were age paired with preterm delivery infants, who had survived in an ex-utero environment, which meant that their development had also taken place outside the uterus. The two groups were assessed on quantitative measures that included molecular markers of granule neuron, purkinje neuron and bergmann glia differentiation, as well as the expression of the sonic hedgehog signaling pathway, that is important for cerebellar growth. We report that premature birth and development in an ex-utero environment leads to a significant decrease in the thickness and an increase in the packing density of the cells within the external granular layer and the inner granular layer well, as a reduction in the density of bergmann glial fibres. In addition, this also leads to a reduced expression of sonic hedgehog in the purkinje layer. We conclude that the developmental program of the cerebellum is specifically modified by events that follow preterm delivery.

## Introduction

The cerebellum is acknowledged as being important not only for motor coordination, but also for abstract mental processes such as thought [1]–[7]. Therefore perturbations in cerebellum development can result in cognitive deficits [8]–[12]. In this regard, several mental disorders have been correlated with cerebellar dysfunction. Cerebellar lesions show clinical symptoms that point to its critical role in mental functions [1], [8], [12], [13]. In humans, a rapid growth in cerebellum development takes place in the third trimester [14], [15]. This is in striking contrast to the development of the cerebellum in the commonly used animal model system, the rodent, in which the cerebellum is relatively immature at birth, and the proliferation of the external granular layer (EGL), the formation of the internal granular layer (IGL) and foliation occur postnatally [16]. MRI studies have shown that this rapid growth in the third trimester is impeded by preterm delivery, where childbirth occurs at a period less than 37 completed weeks of gestation, resulting in a smaller cerebellum [15], [17]–[19].

The incidence of premature birth worldwide is around 10% of all births [20]. The survivors of preterm delivery are at an increased risk for cerebral palsy [21] and a great proportion of them have cognitive, learning and behavioral problems in later life [15], [22], [23]. Given the importance of the cerebellum in cognitive functions, and the rapid phase of development that occurs in the third trimester, there is little information on how the normal developmental program of the cerebellum is modified by change in the environment, due to a preterm delivery. This study addresses the question of how preterm delivery leads to several changes in cerebellar histogenesis, which in turn could possibly account for abnormalities detected in the cerebellum.

The human cerebellum, in-utero, undergoes a clearly defined transition from a 5-layered structure, to a mature and anatomically simpler 3-layered structure, making the study of developmental alterations at the cellular and molecular level in the cerebellum possible [15], [24]. Our aim was to address the issue of how the normal developmental program is perturbed due to premature birth. Our samples (Table S1) were age paired in days and grouped into those that were stillborn infants (0 day ex -utero survival) and premature infants (5–36 days survival ex-utero). Several morphological parameters and molecular markers were analyzed in the cerebellum of preterm infants, who had survived in an ex-utero environment, and compared with age paired infants, in whom cerebellar development had taken place in-utero.

The sonic hedgehog (Shh) signaling pathway is important for granule cell and bergmann glia development [25]. Our previous results [26] suggest that after birth, Shh signaling is downregulated in the human cerebellum, which is correlated with the disappearance of the EGL. Therefore, we investigated whether premature birth could lead to a downregulation of the Shh pathway, leading to a thinning of the EGL layer. The expression of doublecortin was used as a marker for cells that had exited the cell cycle and commenced migration and β-III tubulin was used as a marker for neuronal differentiation [27]. Calbindin and ITPR were used as specific markers for purkinje cell differentiation [28]–[30] and GFAP as a marker for bergmann glia differentiation [31].

This study reports a selective change in the differentiation of granule cells and the bergmann glia and a reduction in Shh signaling due to the ex-utero environment that could have major consequences for later outcomes.

## Results

The summary of the results obtained is presented in Table 1. Samples (Table S1) were age paired in days and grouped into those that were stillborn infants (0 day ex-utero survival) and premature infants (5–36 days survival ex-utero).

**Table 1:** Summary of the results obtained.

Measure	Mean differences between preterm delivery infants and controls adjusted on age in days (Confidence interval 95%)	p
EGL cell density(number/25 µm sq)	1.150 (0.002; 2.300)	0.05
Thickness of the EGL(µm)	−4.069 (−7.470; −0.668)	0.02
Thickness of the ML(µm)	−0.407 (−1.206; 0.391)	0.29
Number of calbindin positive cells (per 100 µm)	5.901 (−13.581; 25.399)	0.53
Number of ITPR positive cells (per 100 µm)	−0.438 (−1.226; 0.349)	0.25
Number of GFAP positive bergmann glia fibres(per 100 µm)	−0.0171 (−0.0002; 0.0003)	0.02
IGL cell density(number/25 µm sq)	2.399 (0.791; 4.007)	0.006

Table 1 - Summary of the results obtained. Mean differences between preterm delivery infants and controls adjusted on age in days for each measure. Abbreviations used - EGL = external granular layer, ML = molecular layer, PL = purkinje cell layer, IGL = internal granular layer, ITPR – inositol tri-phosphate receptor, GFAP – Glial Fibrillary Acidic Protein.

### EGL cell density is increased but EGL thickness reduced during ex-utero development

**Figure 1:**
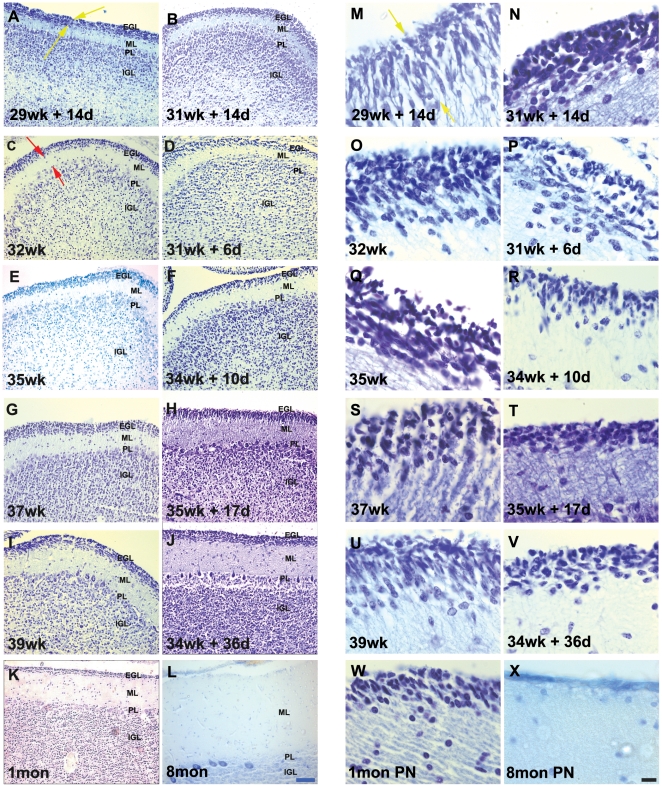
Cresyl violet stained sections of the developing human cerebellum. (A–X) A greater EGL cell density and reduced EGL thickness were reported in preterms with ex-utero exposure, as compared to their age matched stillborn controls. ML thickness was increased in preterms with ex-utero exposure born after 34 weeks gestation, as compared to their age matched controls. Compare E with F, G with H and I with J. Abbreviations used - wk = number of gestational weeks, d = number of postnatal days, mon PN = postnatal months - born at term. EGL = external granular layer, ML = molecular layer, PL = purkinje cell layer, IGL = internal granular layer. Scale bar = 50 µm (A–L) and 20 µm (M–X).

**Figure 2:**
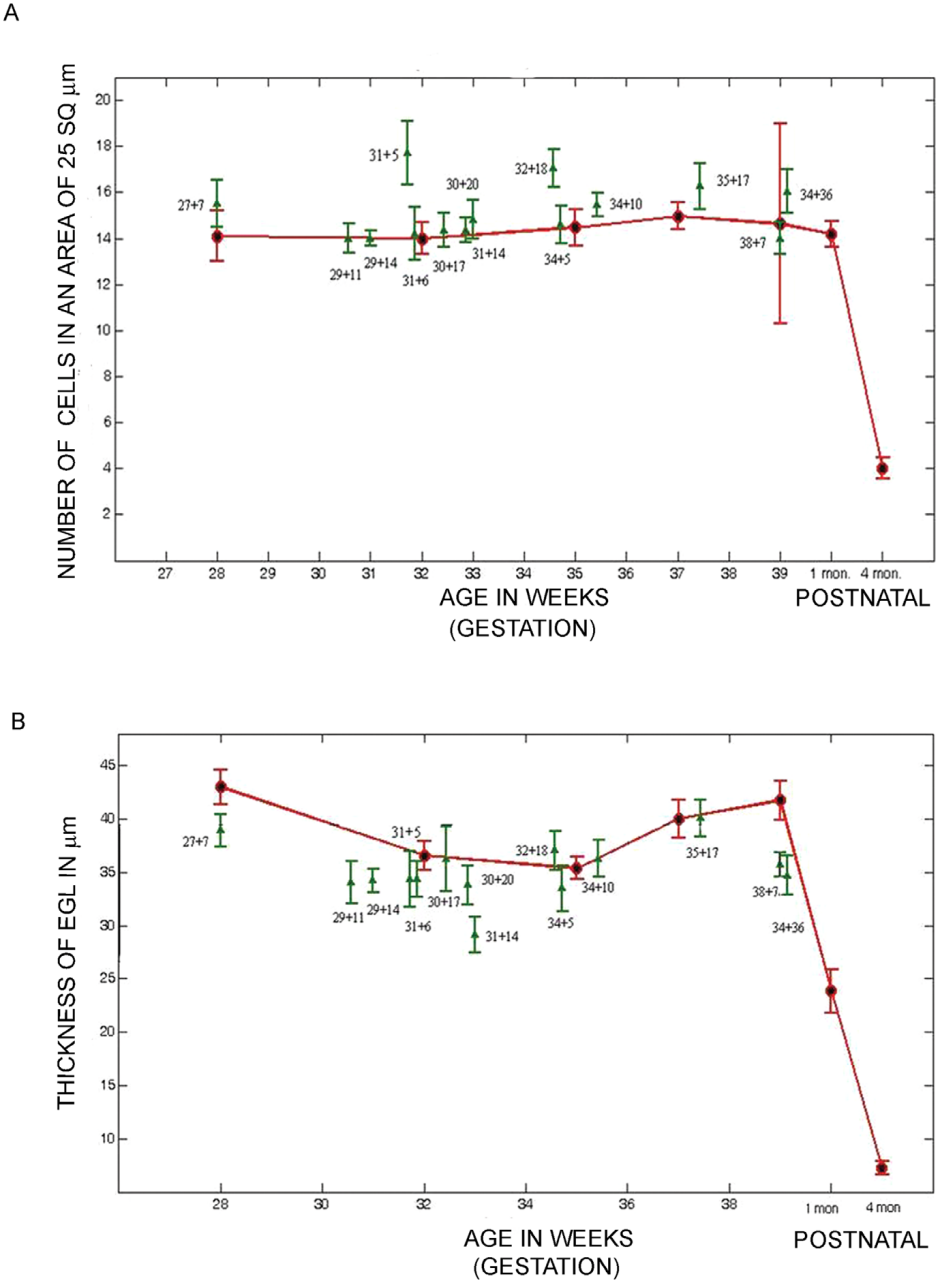
A significant change in EGL cell density and EGL thickness is seen in samples surviving ex-utero. (A) Graph showing the density of cells in the EGL versus age (B) Graph showing the thickness of the EGL versus age. Data is shown as mean ± standard deviation of multiple measurements from single sample (SD). Red = still born and infants that were born at term and survived postnatally (1–8 months). Green = preterms that survived in an ex-utero environment.

Published data of human cerebellum development shows that the cells of the EGL are uniformly packed and the thickness of the layer increases until about 21 weeks of gestation. By the third trimester, it is possible to identify two compartments, whose cellular densities differ from each other. The outer compartment, located close to the pial surface, corresponds to the dense proliferating precursor cell layer, and the inner one corresponds to less densely packed post-mitotic, premigratory neurons. The identification of the EGL was done as indicated in previous studies [14], [24], [32]. The area present between the pial surface and the beginning of the cell sparse region of the ML was used (Figure 1A, yellow arrows). The cell density of the EGL was calculated by counting the number of cells present in a 25 square µm grid. Statistical analysis showed that the cell density in the EGL was significantly higher in specimens that had survived ex-utero, when compared to age matched controls (Figure 1, for example compare O with P, Q with R, S with T and U with V, Figure 2A graph). The thickness of the EGL was calculated by measuring the distance between the pial surface and the beginning of the ML (see Figure 1M, yellow arrows). The thickness of the EGL (Figure 1, for example compare C with D, E with F, G with H and I with J, Figure 2B Graph) was significantly decreased in the specimens that had survived ex-utero. Looking in more detail at the EGL cells of the older specimens, it is observed that although the cell density is considerably reduced, Nissl stained outer EGL in the ex-utero survival infants still persists (Figure 1, compare panels Q and R and panels S and T). Moreover, the inner EGL that normally consists of less densely packed premigratory granule neurons, is not clearly separable in the ex-utero survival samples (Figure 1, compare panels S to T and U to V). This observation is consistent with the significant increase in the average cell density across the EGL in the ex-utero survival samples.

### Thickness of the molecular layer is unchanged during ex-utero development

**Figure 3:**
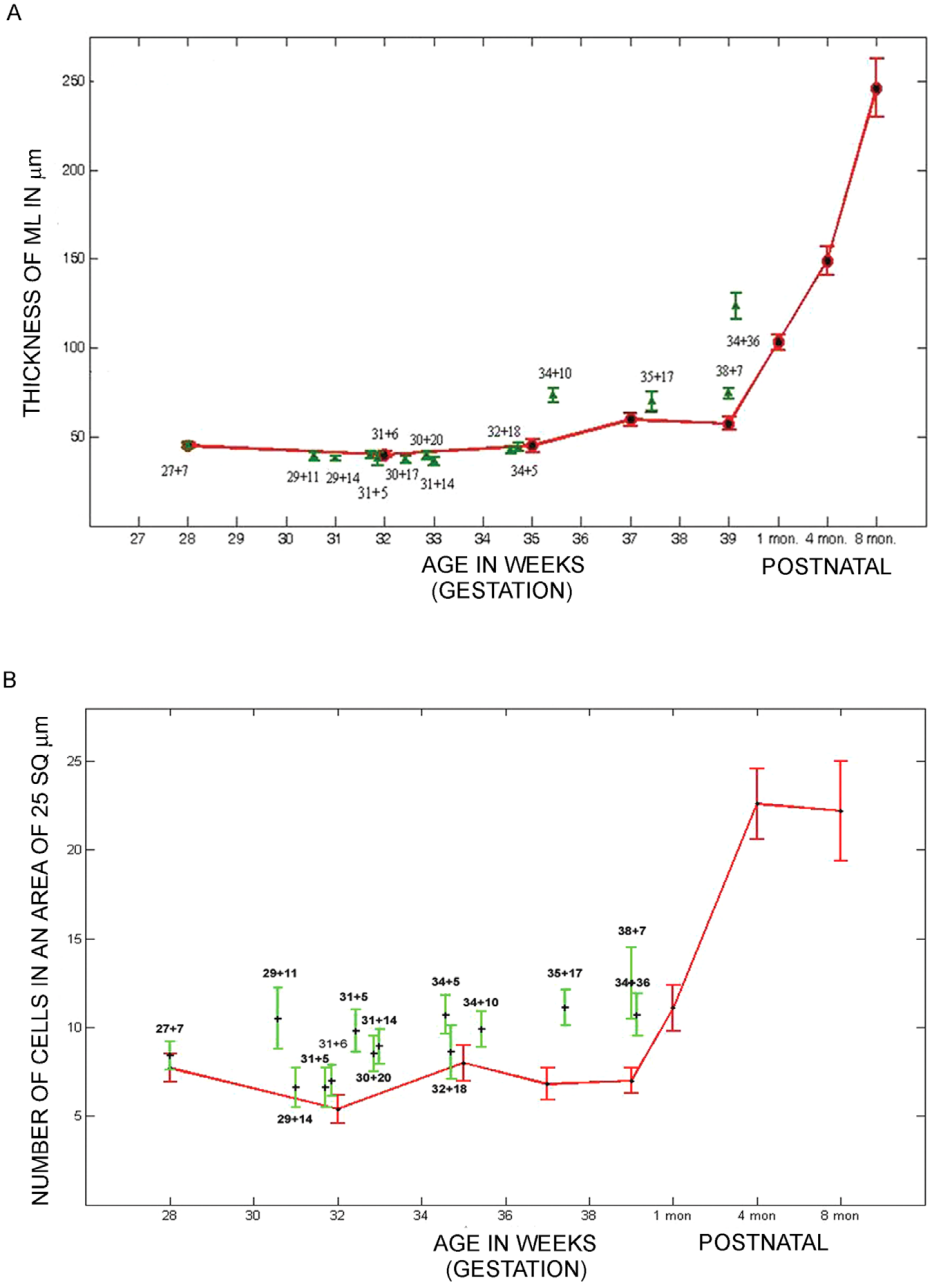
IGL cell density is increased, although there is no significant change in ML thickness in the samples surviving ex-utero. (A) Graph showing the thickness of the ML versus age. (B) Graph showing the density of cells in the IGL versus age. Data is shown as mean ± standard deviation of multiple measurements from single sample (SD). Red = still born and infants that were born at term and survived postnatally (1–8 months). Green = preterms that survived in an ex-utero environment.

The thickness of the molecular layer is contributed by different components of the developing cerebellum at different developmental stages. At 16 weeks, the ML is composed of inwardly migrating granule neurons from the EGL and also cells that are most likely immature interneurons. These cells continue to mature between 21 and 25 weeks and have secondary branches. In addition, the afferent climbing fiber terminals also contribute to the increase in the size of the molecular layer. The molecular layer continues to increase in size as the purkinje cell dendrites develop, and the parallel fibers of the maturing inner granule neurons organize themselves. The width of the molecular layer was analyzed by calculating the distance between the end of the EGL and the PL (See Figure 1C, red arrows). Results showed that there was no statistically significant difference between the widths of the molecular layer in specimens that had survived ex-utero, when compared to age matched controls (Figure 3A, Graph). Interestingly however, although not statistically significant when taking all samples into account, all four samples that were aged greater than 34 gestational weeks and had survived ex-utero, showed an increase in the thickness of the molecular layer (see Figure 3A, graph and Figures 1, compare panel G and I, ML with panel H and J, ML). Taken together with the reduction in the thickness of the EGL at the later ages seen in the ex-utero samples, the increase in the ML thickness seen in these older samples could be a consequence of this reduction.

### Ex-utero environment does not alter the expression of EGL differentiation markers β-III tubulin and doublecortin

**Figure 4:**
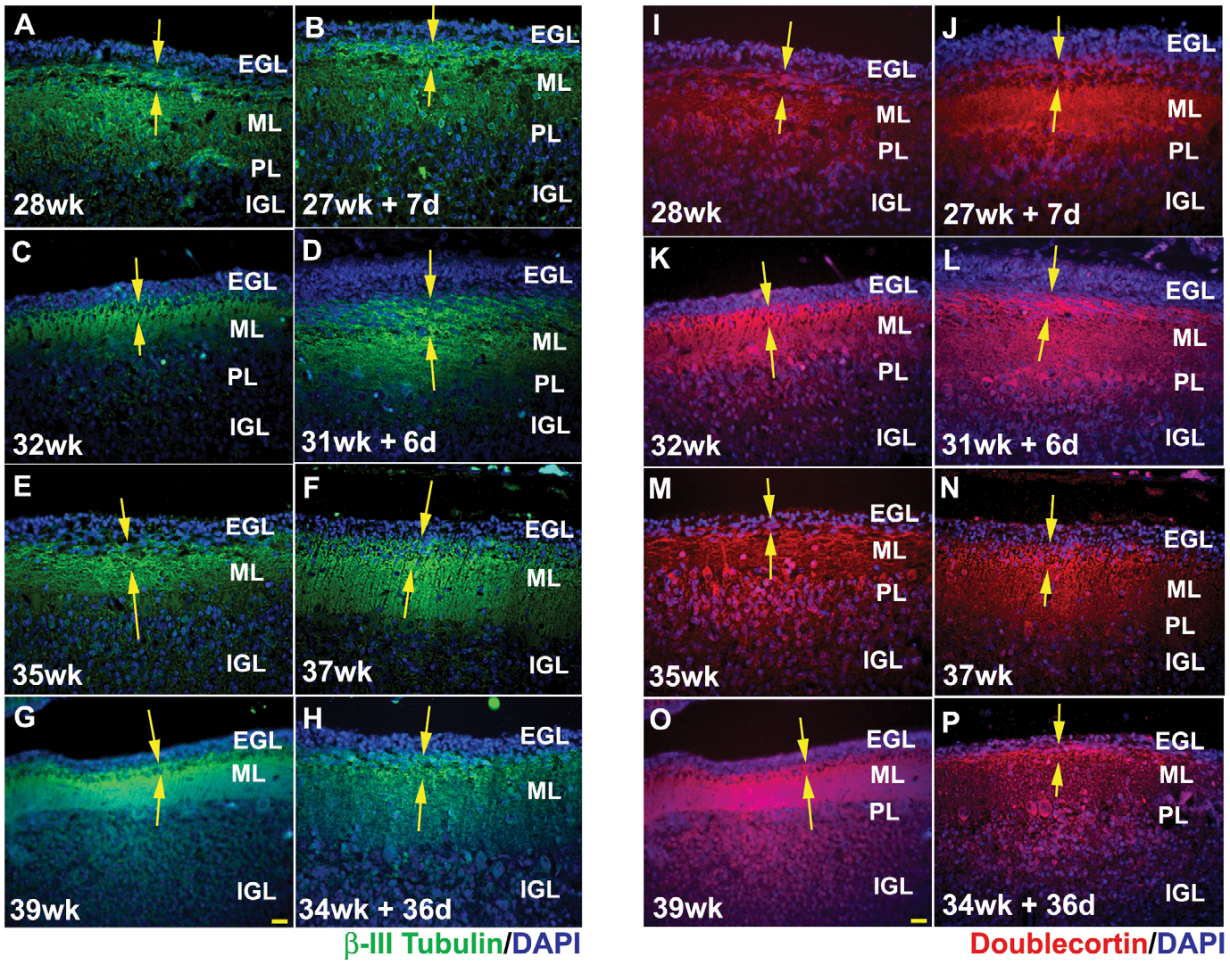
Ex-utero environment does not alter the expression of EGL differentiation markers β-III tubulin and doublecortin. β-III Tubulin (Green) (A–H) and doublecortin (Red) (I–P) positive cells in the inner EGL (yellow arrows). There is no drastic difference seen in the number of β-III Tubulin and doublecortin positive cells in the EGL of stillborn controls and preterms that have survived in an ex-utero environment. DAPI staining was used to get a measure of the total number of cells in the EGL. Blue = DAPI. Abbreviations used - wk = number of gestational weeks, d = number of postnatal days. EGL = external granular layer, ML = molecular layer, PL = purkinje cell layer, IGL = internal granular layer. Scale bar = 50 µm.

To study whether the EGL cells expressed markers of differentiation, the expression of β-III tubulin and doublecortin was examined. Both β-III tubulin (Figure 4, A–H) and doublecortin (Figure 4, I–P) were strongly expressed in cells in the deeper layers of the EGL (yellow arrows). Although the EGL was thinner in the age-matched specimens that survived ex-utero, the percentage of cells expressing β-III tubulin and doublecortin were unchanged in age-matched specimens that had survived ex-utero. Thus, approximately 40% of the EGL population stained positive for β-III tubulin and doublecortin in both groups. This result suggests that the primary effect is not in the ability of cells to express markers of neuronal differentiation and migration. Rather the primary effect based on the Nissl stain figures seems to be a reduced number of cells in the EGL, leading to a reduced EGL especially in later developmental stages.

### Maturation of purkinje cells is not affected by ex-utero development

**Figure 5:**
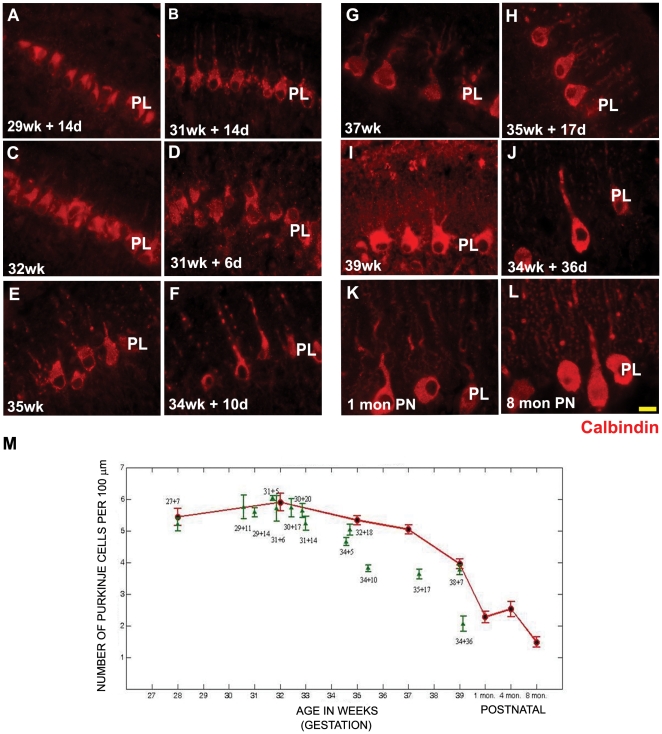
Calbindin positive purkinje cells of the cerebellum. There is no statistically significant difference in the number of calbindin positive purkinje cells of the cerebellum of preterms with ex-utero survival in comparison to their age matched still born controls (A–L). However the purkinje cell number was seen to decrease in preterms with ex-utero exposure, born after 34 weeks gestation, as compared to their age matched controls. The sections have been stained using calbindin antibody raised in rabbit (red). Abbreviations used - wk = number of gestational weeks, d = number of postnatal days, mon PN = postnatal months - born at term PL = purkinje cell layer. Scale bar = 20 µm (M) Graph showing the number of calbindin positive cells in the PL of the human cerebellum versus age. Data is shown as mean ± standard deviation of multiple measurements from single sample (SD). Red = still born and postnatal survival born at term. Green = preterms that survived in an ex-utero environment.

Calbindin D2K was used as a marker for purkinje cells (Figure 5, A–L, Figure S1, A–L). In addition, a second marker inositol-triphosphate receptor (ITPR) was also used to assess the development of purkinje cells (Figure S2, A–X). The PL is easily distinguished due to their large sized cell bodies and characteristic dendritic arborization. The number of calbindin positive or ITPR positive cells were assessed by counting the number of purkinje cell bodies per unit length, in a given field. Statistical analysis showed that on using either calbindin (Figure 5M, graph) or ITPR to mark purkinje cells (Figure S2Y, graph), the number of purkinje cells did not vary significantly between specimens that had survived ex-utero, when compared to age matched stillborn samples. Interestingly however, if one looked at the samples aged 33 g.w and beyond, the purkinje cell number for the ex-utero survival infants, using either of the purkinje cell marker, was lower than their age matched stillborn samples (Figures 5M graph; Figure S2Y, graphs). Since this was especially pronounced only during later stages, it is possible that this measure was not picked out as being statistically significant, when one looked at the trend across all ages.

### GFAP positive bergmann glia fibers are decreased during ex-utero development

**Figure 6:**
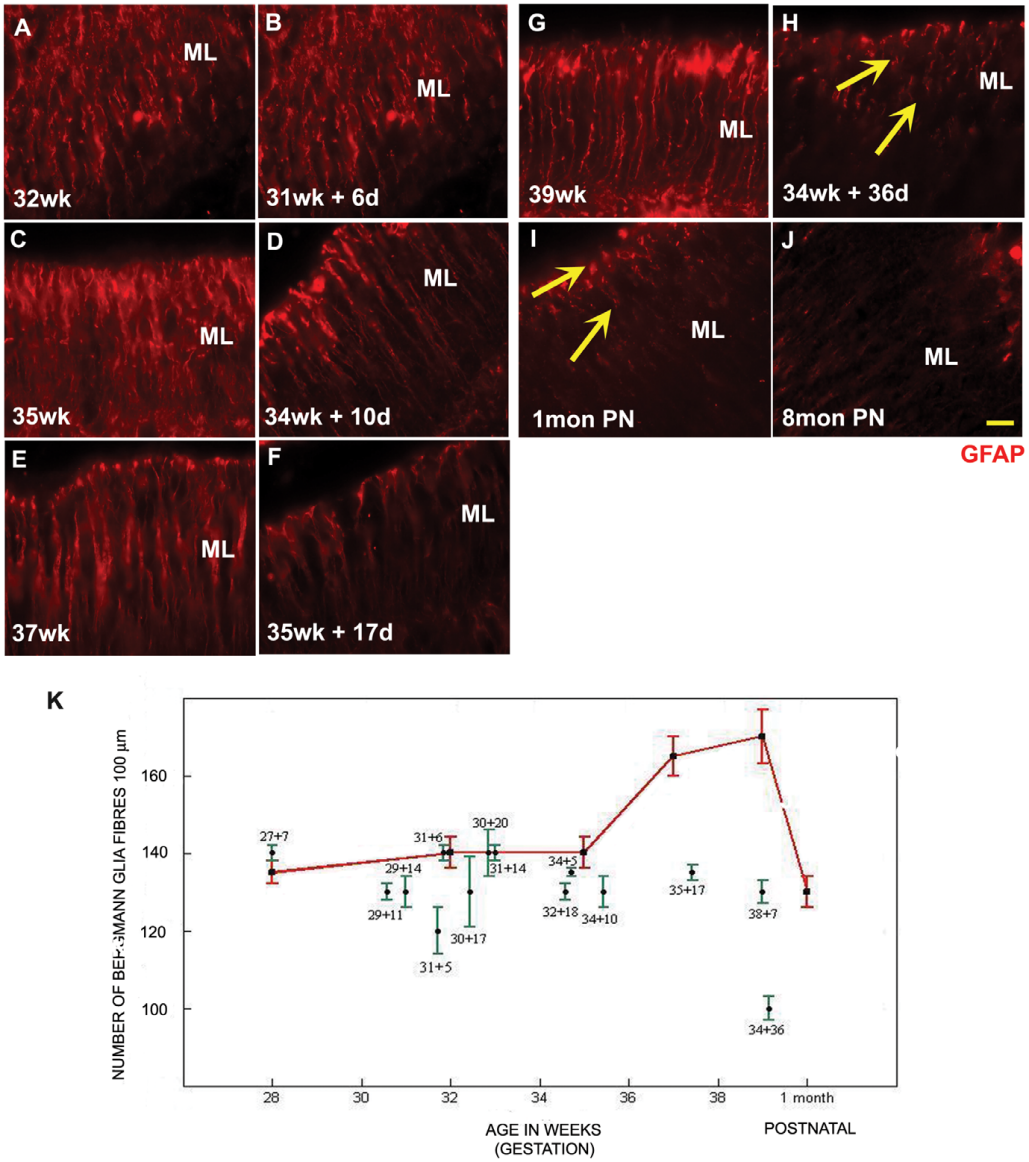
Bergmann glia fibre count is reduced in samples surviving ex-utero. The number of GFAP positive bergmann glia fibres in the ML of the human cerebellum (A–J), was found to reduce in preterms with ex-utero survival in comparison to their age matched still born controls. The morphology of the fibres from the individual with maximum ex-utero exposure (34 g.w+36 d) was found to be similar to those seen in a 1 month postnatal cerebellum in an individual born at term; Compare H with I, arrows. Abbreviations used - wk = number of gestational weeks, d = number of postnatal days, PN = postnatal months- born at term, PL = purkinje cell layer. The sections have been stained using GFAP antibody raised in rabbit (red). Scale bar = 20 µm (K) Graph showing the number of GFAP positive bergmann glia fibres in the ML of the human cerebellum versus age. Data is shown as mean ± standard deviation of multiple measurements from single sample (SD). Red = still born and postnatal survival born at term. Green = preterms that survived in an ex-utero environment.

The cells of the EGL migrate in an outside-in manner with the aid of the fibers of bergmann glia, to form the IGL. The presence of these fibers is hence an indication of an ongoing process of neuronal migration in the cerebellum. The number of GFAP positive glial fibers (Figure 6 A–J; Figure S3, A–J) was analyzed by counting the number of fibers that intersected the line drawn parallel to the PL in a given field. The fibers were counted at the level of the molecular layer, since the glial fibers tended to branch at the level of EGL and the IGL. Statistical analysis showed that the number of GFAP positive fibers was significantly reduced in the specimens that had survived ex-utero. One sees a consistent decrease in GFAP positive fibre number from 34 weeks onwards in the ex-utero surviving infants when compared to their age-matched stillborn controls (Figure 6 and Figure S3, for example compare C with D, E with F and G with H, 6K graph). The fibre morphology of the specimens aged 34 weeks with 36 days of ex-utero exposure, was similar to that of a 1 month neonatal brain born at term, in that the fibres did not span the length of the ML and seemed to break abruptly (Figure 6 and Figure S3, compare H with I, arrows).

### The expression of sonic hedgehog (Shh) and its downstream effectors is decreased during ex-utero development

**Figure 7:**
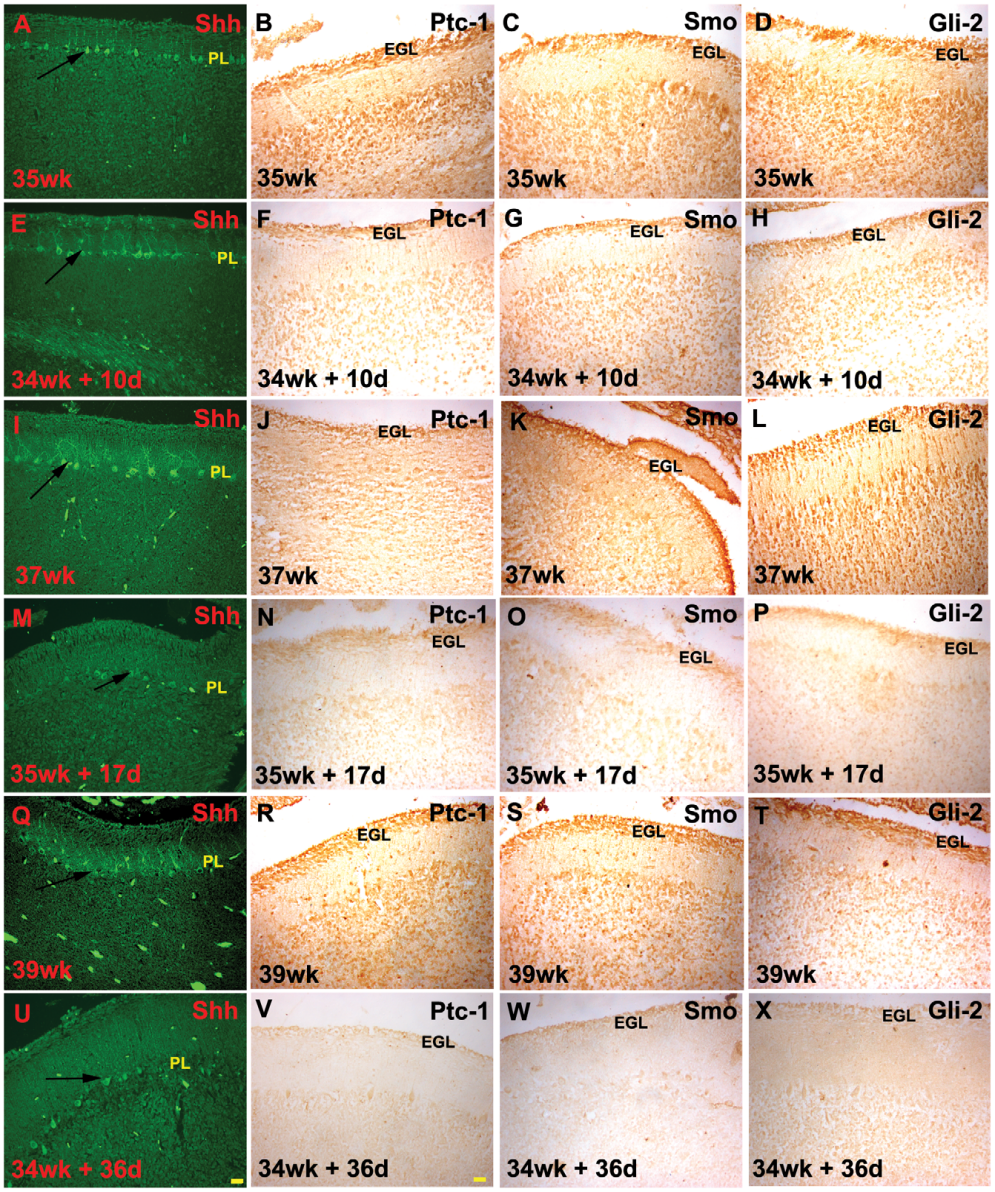
Distribution of sonic hedgehog (Shh), patched-1 (Ptc), smoothened (Smo) and Gli-2 across ages. The levels of Shh, its receptor Ptc and its downstream effectors were seen to decrease in preterms with maximum ex-utero exposure, in comparison to their age matched controls. Compare 37 wk (I–L) with 35 wk+17 d (M–P) and 39 wk (Q–T) with 34 wk+36 d (U–X); Abbreviations used - wk = number of gestational weeks, d = number of postnatal days. EGL = external granular layer, ml = molecular layer, PL = purkinje cell layer, IGL = internal granular layer. Sections were stained by immunofluorescence for Shh, while Ptc, Smo and Gli-2 reactivity were developed using the peroxidase method. Scale bar = 50 µm.

In the developing human cerebellum, Shh is expressed strongly by the purkinje cells during gestation. However this expression decreases postnatally [26]. We analyzed the levels of expression of Shh, its receptor patched (Ptc), smoothened (Smo) and its effector Gli-2 by comparing staining intensities across specimens (Figure 7, A–X, Table S2). We found that in the premature birth samples with ex-utero development, the levels of Shh and its downstream signaling effectors was dramatically reduced, when compared to their age matched stillborn controls. Figure 7 shows the expression of Shh in premature birth samples with varying ex-utero survival days (Figure 7E, Figure 7M and Figure 7U) as compared to their age matched control (Figure 7A, Figure 7I and Figure 7Q). The expression level of Ptc (Figure 7F, Figure 7N and Figure 7V), Smo (Figure 7G, Figure 7O, and Figure 7W) and Gli-2 (Figure 7H, Figure 7P and Figure 7X) was also reduced in preterm delivery samples compared to age matched stillborn controls (Figure 7B, Figure 7J and Figure 7R for Ptc, Figure 7C, Figure 7K, and Figure 7S for Smo, Figure 7D, Figure 7I, and Figure 7T for Gli-2).

### Proliferation rate coincides with reduction in Shh signaling

**Figure 8:**
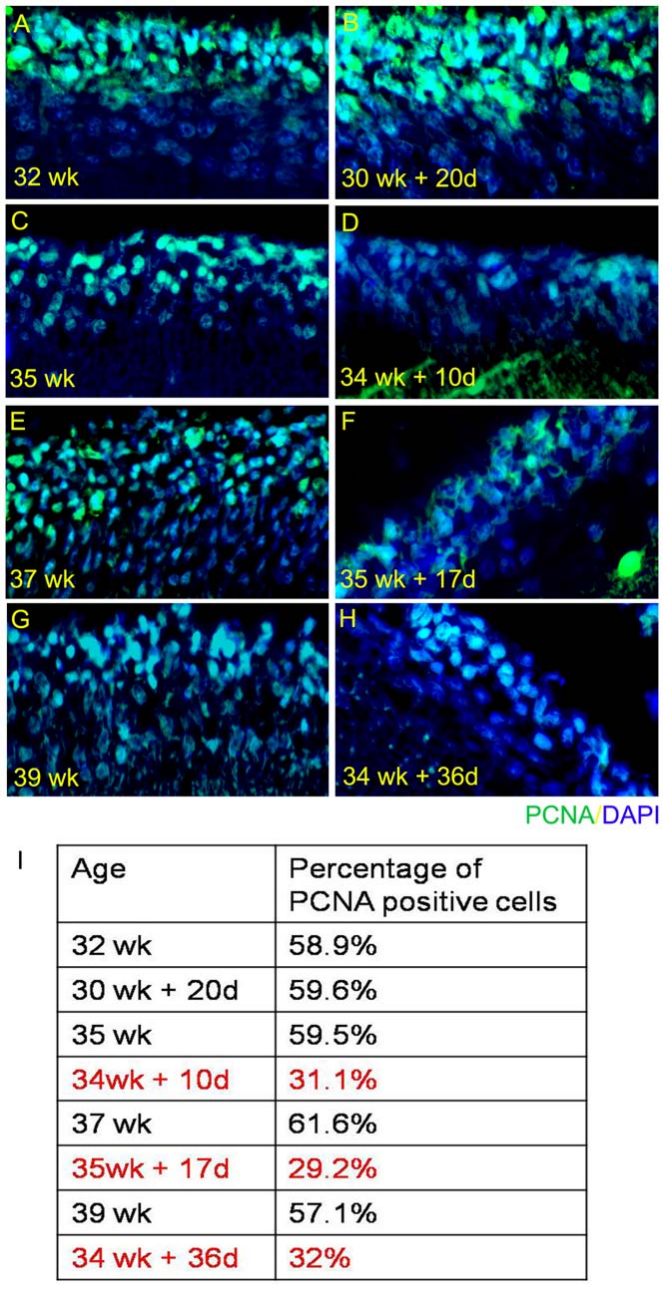
The number of PCNA positive cells decreases with reduction in Shh signalling. While we find that there is no major difference in the number of proliferating population between (A) 32 weeks and (B) 30 wk+20 d, there is a significant difference in the preterms with reduced Shh signalling. For example, compare (C) 35 wk with (D) 34 wk+10 d; (E) 37 wk with (F) 35 wk+17 d; and (G) 39 wk with (H) 34 wk+36 d. Sections were stained by immunofluorescence for PCNA (green) and mounted using vectashield DAPI (blue).

Shh is a potent mitogen and is one of the major signals for proliferation of the granule cell precursors of the EGL (25). We find that the reduction in Shh signaling shown in Figure 7 coincides with a reduction in proliferation of GCPs, identified by PCNA immunohistochemistry (Figure 8). In comparison to 35, 37 and 39 week stillborn controls (Figure 8A,C,E) wherein approximately 60% of the EGL cells were labeled with PCNA the age matched premature samples (Figure 8B,D,F) had an approximately 50% reduction in the number of PCNA labeled cells (Figure 8I, Table).

### IGL cell density is increased during ex-utero development

The consequences of EGL development on the IGL formation was studied by analyzing the cell density of the IGL. This was calculated by counting the number of cells present in a grid of 25 square µm, in the area that was situated below the PL and above the white matter region. There was a statistically significant difference in the densities in specimens that had survived ex-utero when compared to age matched stillborn samples (Figure 1, for example compare C with D, E with F, G with H and I with J, Figure 3B, graph). As expected, the IGL cell density was seen to increase with age (Figure S4, A–L; Figure 3B, graph), and this increment was tremendous, postnatally as the brain matured and the EGL disappeared (Figure S4, K–L).

## Discussion

This study describes for the first time changes that occur at the cellular level and suggests potential mechanisms for perturbations in cerebellum development that occur after preterm delivery. The main findings of this study are that specific cell types in the cerebellum are affected, rather than generalized changes during the period of ex-utero development, following preterm delivery. To summarize, the EGL thickness is reduced, packing density of the EGL cells is increased, and the number of bergmann glial fibers are reduced when development occurs after preterm delivery, in an ex-utero environment. In contrast, the density of the purkinje cell layer and the thickness of the molecular layer are unaffected. In addition, there is a reduction in Shh signaling when ex-utero development follows preterm delivery, compared to their age-matched stillborn controls.

Before discussing the finding, one would like to state the difficulties and drawbacks inherent in such a study, yet impress the validity and need for such analyses. Firstly, it is clear that the stillborn births are not a normative sample and the age equivalent stillborns are few in number. Despite the fact that the controls may have suffered brain insults, our comparison with prematurity suggests that prematurity by itself may be sufficient to cause disruptions to the cerebellar developmental program. Secondly, it is clear that the premature birth group, that survived for varying times after birth is a heterogeneous group with variable number of survival days. However, to take into account these many random variables, we used rigorous statistical modeling. Despite these limitations, the study is validated by the following observation – Firstly when we compared the values that were obtained from stillborn infants at various stage of maturity and compared them to values from previously published studies, the values compared favorably with such normative data (Table S3). Secondly, all parameters that were measured in the cerebellum were not globally affected. Instead specific defects were seen, which were consistent with one another. This would suggest that even though there were variables in the sample beyond control, the method of analysis and the statistics used could pick out the significant trends across these random variables. Thirdly, it is possible that the change that we see in the cases where part of the developmental time has been spent ex-utero, is due to other pathologies such as resuscitation procedures, infection and inflammation, and not due to the environment affecting the development. It is known that inflammation, especially in the newborn, can lead to white matter damage as well as to excitoxic cell death [33], [34]. However it has not been reported that such systemic inflammation can cause the down-regulation of specific signaling pathways. We present a table (Table S4) where the various causes of death have been given and the cases classified accordingly. It is seen that out of the 19 preterm and still born cases that we have analyzed only 6 have also had sepsis, others have died of other causes. Finally, it must be emphasized that despite these limitations these studies are important because they give rise to novel results and hypothesis on human brain development that cannot be obtained by any other means and that can then be tested by more controlled approaches.

This study is mainly based on immunohistochemical evidence. There is no previous data on the expression of the Shh pathway in the developing human cerebellum. However, the specificity of the staining profiles was assessed by comparison of their expression pattern in the human cerebellum, to that characterized in the developing mouse cerebellum [26]. In the mouse cerebellum, Shh expression is seen in the purkinje cell layer at the time of EGL cell proliferation that occurs postnatally in the mouse [25], [35]. The receptors for Shh, Ptc and Smo are strongly expressed in the granule cell precursors in the EGL [25], [36]. Western blot of the developing human cerebellum indicates the expression of both Shh and its receptor Ptc (Figure S5). In addition, the downstream transcription factors Gli1 and Gli2 are also strongly in the EGL as well as are seen in deeper layers of the cerebellum [37], [38].

Essentially, a similar pattern of expression was observed in the current study in the developing human cerebellum coinciding with the time when a rapid expansion of the EGL was occurring, which in humans, occurs in-utero. The conservation of the expression pattern of the Shh signaling pathway across species validates our approach to assessing the effect of preterm delivery on this important signaling pathway. To assess whether the changes in the EGL seen in this study are due to perturbations in specific aspects of the granule neuron developmental program, i.e., proliferation of precursors vs. migration and differentiation of granule neurons, the expression of a proliferation marker PCNA and two markers of EGL differentiation that are expressed in the inner layers of the EGL were analyzed [39]. There was no qualitative difference between the ex-utero and the age matched stillborn specimens in the expression of doublecortin and β-III tubulin. Since these mark granule cells that have exited the cell cycle and have begun to migrate, this suggests that the thinner EGL may be due to the ex-utero environment specifically affecting the proliferation of EGL cells. This was evinced in the reduced number of cells in the cerebellum of premature births that were labeled with PCNA. The possible reason for this developmental perturbation was next investigated. In rodent studies it has been shown that a reduced number of EGL cells can be the result of a lack of mitogenic signals, the main one being Shh that is secreted by the purkinje cells [16], [25]. The present study shows that there was a clear qualitative difference that was found in the expression of Shh in the purkinje cells in the ex-utero samples when compared to the stillborn age matched controls. Accompanying this decrease in Shh expression there was also a decrease in expression of its receptor complex Ptc-1/Smo and its downstream effectors Gli-1 and Gli-2. Earlier studies in other species have shown that Shh function is required not only for proliferation of the EGL but also for bergmann glia maturation [25]. If indeed there is a decrease in Shh signaling that takes place after birth one would expect that the bergmann glial fibers also to be affected. Consistent with this, the present study shows a statistically significant decrease in the number of bergmann glia fibres in the ex-utero specimens that mirror the decrease in the Shh signaling components. The downregulation of Shh signaling in humans in an ex-utero environment is interesting since this is fundamentally different from what occurs in rodents. In rodents, Shh signaling from the purkinje cells is highest in the first two postnatal weeks, when maximum proliferation of granule cells takes place.

Finally, it must be noted that the increase in the thickness of the molecular layer, the maturation of the purkinje cells, and the migration of the granule cells all significantly vary with fetal age. Therefore, we suggest that these variables are part of an intrinsic developmental program and will not be affected by premature birth and ex-utero development as is observed in the current study. In contrast, the thickness of the EGL and the bergmann glia fibers remains fairly constant during the rapid phase of cerebellar expansion and suggest that these variables are subject to control by extrinsic cues and therefore these variables are likely to be affected by ex-utero development, as observed. It is at present not clear whether all cases of cerebellar volume reduction would show similar underlying pathology and this question awaits further investigation. However, [15] does suggest that the EGL is particularly vulnerable to several types of insults.

In conclusion, this study quantitatively analyzes the changes in cerebellar cortical layers in preterm infants and in addition provides a hypothesis for understanding the reason behind the cerebellar volume change that has been seen in preterm infants. The changes that we observe could be the sequelae that follows premature birth and not due to development outside the womb. Nevertheless it suggests that some aspects of cerebellar histogenesis may be intrinsically driven whereas others are more subject to extrinsic cues. We hope that one can now begin to address the issue of how the environment is able to modulate signaling pathways that govern cerebellar histogenesis and find ways of intervention for the benefit of the preterm infant.

## Materials and Methods

### Tissue

Specimens used were collected in accordance with the guidelines laid down by the Institutional Human Ethics Committee, National Brain Research Centre, Manesar, which have been framed by the Indian Council of Medical Research, India. This study was carried out only after ethical clearance. A detailed account of all the cases used has been provided in Table S1. We obtained cerebellum aged 28 weeks – 8 postnatal months, from the Department of Pathology, All India Institute of Medical Sciences, New Delhi. These were from the fetuses of elective or spontaneous abortions and intra uterine fetal deaths. We also obtained tissue from fetuses that died postnatally. In both cases a written consent was taken from the parents before the specimen were collected. The ethics committee approved of the procedure. All postmortems and subsequent dissection of organs were carried out within 24 hours of death. The tissue had been characterized for presence of brain damage by histopathology. By this criteria of the 19 preterm and still born specimens analyzed, 2 had brain pathology. One of these was a germinal matrix hemorrhage with intraventricular extension and the other had agenesis of corpus callosum and gray matter heteropia. Since no cerebellar pathology was detected these were included in the analysis (see Table S1). The study excluded cases where autolysis of cells or tissue degradation was detected due to intra uterine death. In only one (39 weeks) of the five cases did the fetus die in-utero. Data from this case did not show any evidence of autolysis and tissue degradation. 4 of our 5 controls were live births and died shortly after birth. The cases we have included are those in which the autopsy indicated minimum or no damage to the brain and cerebellum in particular. The tissue was fixed in 4% PFA (pH 7.6) for two weeks, and embedded in paraffin. The cerebellar blocks for the samples used were acquired from the hospital. Sagittal sections of 5 µm thickness were cut using a microtome of the entire block (Leica RM 2135, Germany) and placed on Superfrost Plus white slides (VWR international, USA) that were subbed using Poly-L Lysine (Sigma, USA). Slides were stored at room temperature until immunostaining was performed. We would like to reiterate that the entire process of tissue collection, obtaining consents, and subsequent experimentation, was approved by the institutional human ethics committee.

### Histochemical analysis

Immunohistochemistry was performed on serial sections. Paraffin sections were dewaxed and hydrated before staining. Sections were quenched with 3% hydrogen peroxide to remove endogenous peroxidase activity. This step was skipped for sections on which immunofluorescence was carried out. 0.5% Triton X was used to permeabilize sections. To reduce non-specific binding, sections were blocked with 5% serum using serum from host species of the secondary antibody. 5% BSA was used for blocking in co-localization studies. Sections were incubated in primary antibody for 16–48 hours at 4°C at optimum dilutions in a humid chamber. All primary and secondary antibodies used in this study have been listed in Table S5. Slides treated for immunoflourescence were mounted in Vectashield mounting media with DAPI (Vector laboratories, USA), while those treated with biotinylated secondary antibody were incubated in the ABC reagent, (1∶50, Avidin-Biotin complex reagent; Elite ABC kit, Vector laboratories, Burlingame, CA, USA) and developed using the Nova Red Kit (Vector Laboratories, Burlingame, CA, USA). They were air dried overnight and cover-slipped with DPX mounting medium. For each antibody, one section was used as a negative control where in the section was incubated with all the above solutions except the primary antibody.

For Nissl staining, sections were stained with 1% thionin solution for 1 minute and then differentiated in a solution containing 1∶1 ethanol and dioxane (Merck, India). Sections were dehydrated in Dioxane for 5 min, followed by immersion in xylene and were then cover-slipped with DPX mounting medium.

### Western Blotting

Radioimmunoprecipitation assay buffer (RIPA) was added to human and mouse tissue (for 1 mg of tissue, 100 µl of buffer was added) and homogenised. The homogenised mixture was subjected to centrifugation. The supernatant (whole protein extract) was collected. The protein concentration was estimated using Bradford's reagent and 60 µg of protein was electrophorosed on 7–10% polyacrylamide gel. The proteins on the gel that were transferred to PVDF membrane were probed with primary antibody overnight – Shh (Santa Cruz, 1∶1000), Ptc (Abcam, 1∶1000). After appropriate washes with 1X TBST the blots were probed with secondary antibody (Vector laboratories) for two hours. Finally, the blots were washed with 1X TBST and developed using chemiluminescent reagent (Amersham) and exposed to Chemigenius, Bioimaging System (Syngene UK) for developing. Images were captured using Genesnap software (Syngene).

### Microscopy

All bright field images were captured using Leica DFC 320 (Germany) and analysed using the Leica IM50 software. Fluorescent Images were captured on a Zeiss Axioplan II (Germany) microscope using an Axio Cam HRC camera and analysis was done using the Zeiss KS 400 3.0 software. Apart from minor adjustment of contrast and brightness no additional image alteration was done.

### Histological analysis

Blinded counts and scoring methods were used throughout the study. EGL and IGL cell density analysis was done on a total of 3 sections with 20 observations per section using Neurolucida system (MBF Bioscience, Microbrightfield Inc. USA) linked to an Olympus BX51 microscope (Japan) and camera (Olympus U-TV1X-2, Japan). The cell number in a total of 20 grids of area 25sq µm was counted, per section. The area between the crowns and depths of the cerebellar folia were chosen for measurements as the thickness and basic cytoarchitecture here is more comparable across different cerebellar regions. Thickness of the EGL and the ML, purkinje cell counts and GFAP fiber counts were done using IM50 software (Leica, Germany). For all of the above measurements, 3 sections from each specimen were analyzed. For EGL and ML thickness, 20 and 10 observations were made per section respectively, at 20 different locations. A total of 9 observations were made for purkinje cell number, bergmann glia fibre number, β- III tubulin positive and doublecortin positive cells. Here 9 different fields were captured for measurement.

For the purkinje cell number, the number of cells present on the line that intersected the PL in a given field were counted while in case of the bergmann glia fibre count, the number of fibres present above the line that intersected the PL in a given field were counted.

For calculating the percentage of β- III tubulin positive and doublecortin positive cells, the number of DAPI stained nuclei in that field were counted and used as a measure of total cells. Three individuals were involved in the quantitation and they were blinded to the identity of the specimen. The count for total number of proliferative cells by PCNA staining was done in a manner similar to that mentioned above.

### Analysis of intensities of staining of Shh and its downstream factors

All images were captured at the same intensity of exposure and were subsequently coded. A blind approach was used to evaluate intensities of staining (Table S2). We used calbindin as an internal control to compare the changes in the intensity of staining. Negative controls were included with each staining experiment. We used a 0–3 intensity scale to describe the intensity of staining with 0 indicating no staining while 3 indicated highly intense staining.

### Graphs

All graphs were constructed using the MATLAB program.

### Statistics

The quantitative data obtained were described by the median and extreme values. The modelling consisted of finding a relationship between the type of measure (for example EGL thickness) and prematurity or stillbirth adjusted for age in days (27 weeks in-utero+7 days ex-utero = 196 days, 28 weeks stillbirth = 196 days). The population consisted of 13 cases of preterm delivery with variable survival times and 5 cases of stillborn. Each case had measurements from multiple sections and within each section many areas were sampled. A three level nested linear model also known as unconditional hierarchical nested linear model was used where the measure (for example EGL thickness) was nested in the slice and the slice was nested in the observation. Thus the three levels are level 1: individual measurements, level 2: cerebellum sections and level 3: type of measure. Assumptions of the model such as residual normality and absence of heteroscedasticity were checked. All tests were bilateral and statistical significance was set to 5% that is CI = 95%. Analyses were performed with SAS 9.1 (SAS Institute Inc., Cary, NC).

The hierarchical nested linear model is represented as
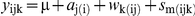
where

*a*_j(i)_ is the effect of the observation i that is associated with prematurity j

*w*_k(ij)_ is the effect of the section k that is associated with the observation i associated with prematurity j

*s*_m(ijk)_ is the effect of the measure m associated with the section k that is associated with the observation i associated with prematurity j.

## Supporting Information

Figure S1 **Calbindin positive purkinje cells of the cerebellum.** (A–L) The sections have been stained using calbindin antibody raised in rabbit (red). Scale bar = 50 µm. Abbreviations used - wk = number of gestational weeks, d = number of postnatal days, mon PN = postnatal months - born at term. PL = purkinje cell layer.
(TIF)

Figure S2 **Inositol triphosphate receptor (ITPR) positive purkinje cells in the cerebellum.** This was done in order to validate the results obtained using calbindin. (A–X). The sections have been stained using ITPR antibody raised in rabbit (red). Scale bar = 50 µm (A–L), 20 µm (M–X). (Y) Graph showing the number of ITPR positive cells in the PL of the human cerebellum versus age. Data is shown as mean ± standard deviation (SD). Red = still born and postnatal survival born at term. Green = preterms that survived in an ex-utero environment. Abbreviations used - wk = number of gestational weeks, d = number of postnatal days, mon PN = postnatal months- born at term, PL = purkinje cell layer.
(TIF)

Figure S3 **GFAP positive bergmann glia fibres in the ML of the human cerebellum.** (A–J) The sections have been stained using GFAP antibody raised in rabbit (red). Scale bar = 50 µm. Abbreviations used - wk = number of gestational weeks, d = number of postnatal days, mon PN = postnatal months- born at term, ML = molecular layer.
(TIF)

Figure S4 **Cell density in the internal granular layer (IGL) increases in samples surviving ex-utero.** Abbreviations used - wk = number of gestational weeks, d = number of postnatal days, mon PN = postnatal months- born at term. The sections (A–L) have been stained using Cresyl violet. Scale bar = 20 µm.
(TIF)

Figure S5 **Western blot done for sonic hedgehog, and patched, on human and mouse tissue.**
(TIF)

Table S1 **List of all the cases used in the current study.** The stillborn cases (0 day ex -utero survival) are indicated in red; premature infants (5–36 days survival ex-utero) are indicated in green; postnatal months born at term (1,4 and 8 postnatal months) indicated in blue. Abbreviations used - SVD – spontaneous vaginal delivery; Em LSCS – Emergency Lower segment caesarean section; B and M – bag and mask; NICU – Neonatal Intensive Care Unit; CCF – Congestive Cardiac Failure; Non reactive NST – Non Reactive Non stress test; PROM – Premature rupture of membranes; Vent – Ventilator, VD – vaginal delivery; IPPV – Intermittent Positive Pressure Ventilation; ET – exchange transfusion; Rh – rhesus isoimmunisation; SIMV- Synchronised Intermittent Mandatory Ventilation; CPAP – Continuous positive airway pressure; BT – blood transfusion; PIH – Pregnancy induced hypertension; TPN – Total Parenteral Nutrition; ANA - anti nuclear antibody; IUGR – Intra Uterine Growth Retardation; GCMF – gross congenital mal formation; IUD – Intra Uterine Death; NA - Not apparent, PM - Post mortem, Cause of death 1a/b/c have been mentioned as per international classification of diseases (ICD) codes. Cause of death 2 indicates other pathologies that did not directly contribute to death. (-) in Apgar score indicates non availability of data in the particular case.
(DOC)

Table S2 **Intensities of staining of sonic hedgehog, calbindin (internal control), patched, smoothened, Gli-1 and Gli-2 in stillborn and preterm infants.** A double blind approach has been used for the same. A 0–3 intensity scale to describe the intensity of staining with 0 indicating no staining while 3 indicated highly intense staining.
(DOC)

Table S3 **The table lists all EGL and ML thickness values that were obtained from stillborn infants at various stage of maturity in comparison to the values from previously published studies.**
(DOC)

Table S4 **List of all the cases that were used in the current study that have been classified according to their cause of death.**
(DOC)

Table S5 **List of all primary and secondary antibodies used in the study, including their type, dilution and company name.**
(DOC)
