# Supplementary figures and images for: Preterm Delivery Disrupts the Developmental Program of the Cerebellum
Source: PLoS One. 2011 Aug 17;6(8):e23449. doi: 10.1371/journal.pone.0023449 (PMC3157376; doi:10.1371/journal.pone.0023449)

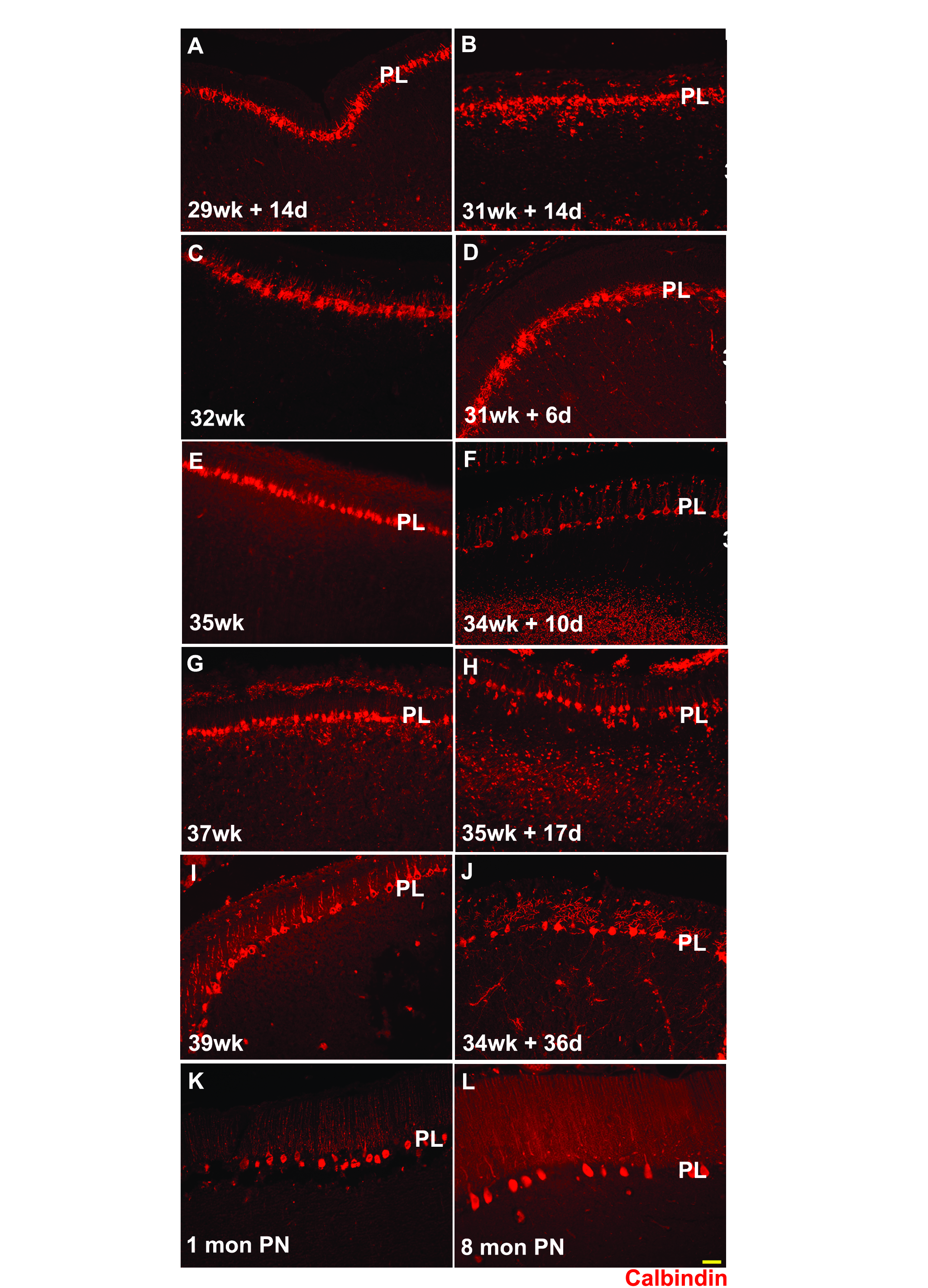

Supplement: Figure S1 — Calbindin positive purkinje cells of the cerebellum. (A–L) The sections have been stained using calbindin antibody raised in rabbit (red). Scale bar = 50 µm. Abbreviations used - wk = number of gestational weeks, d = number of postnatal days, mon PN = postnatal months - born at term. PL = purkinje cell layer. (TIF) [file pone.0023449.s001.tif]

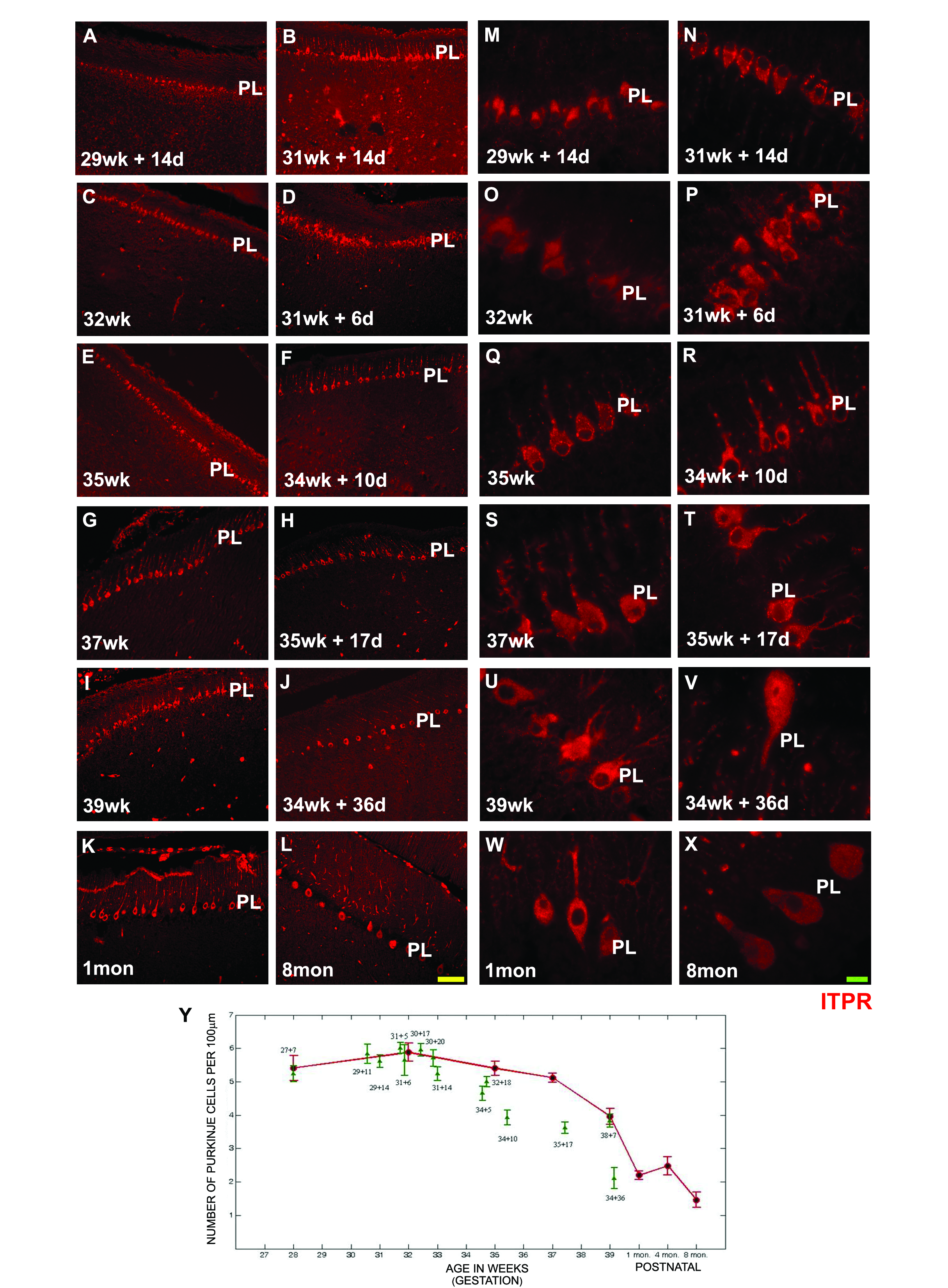

Supplement: Figure S2 — Inositol triphosphate receptor (ITPR) positive purkinje cells in the cerebellum. This was done in order to validate the results obtained using calbindin. (A–X). The sections have been stained using ITPR antibody raised in rabbit (red). Scale bar = 50 µm (A–L), 20 µm (M–X). (Y) Graph showing the number of ITPR positive cells in the PL of the human cerebellum versus age. Data is shown as mean ± standard deviation (SD). Red = still born and postnatal survival born at term. Green = preterms that survived in an ex-utero environment. Abbreviations used - wk = number of gestational weeks, d = number of postnatal days, mon PN = postnatal months- born at term, PL = purkinje cell layer. (TIF) [file pone.0023449.s002.tif]

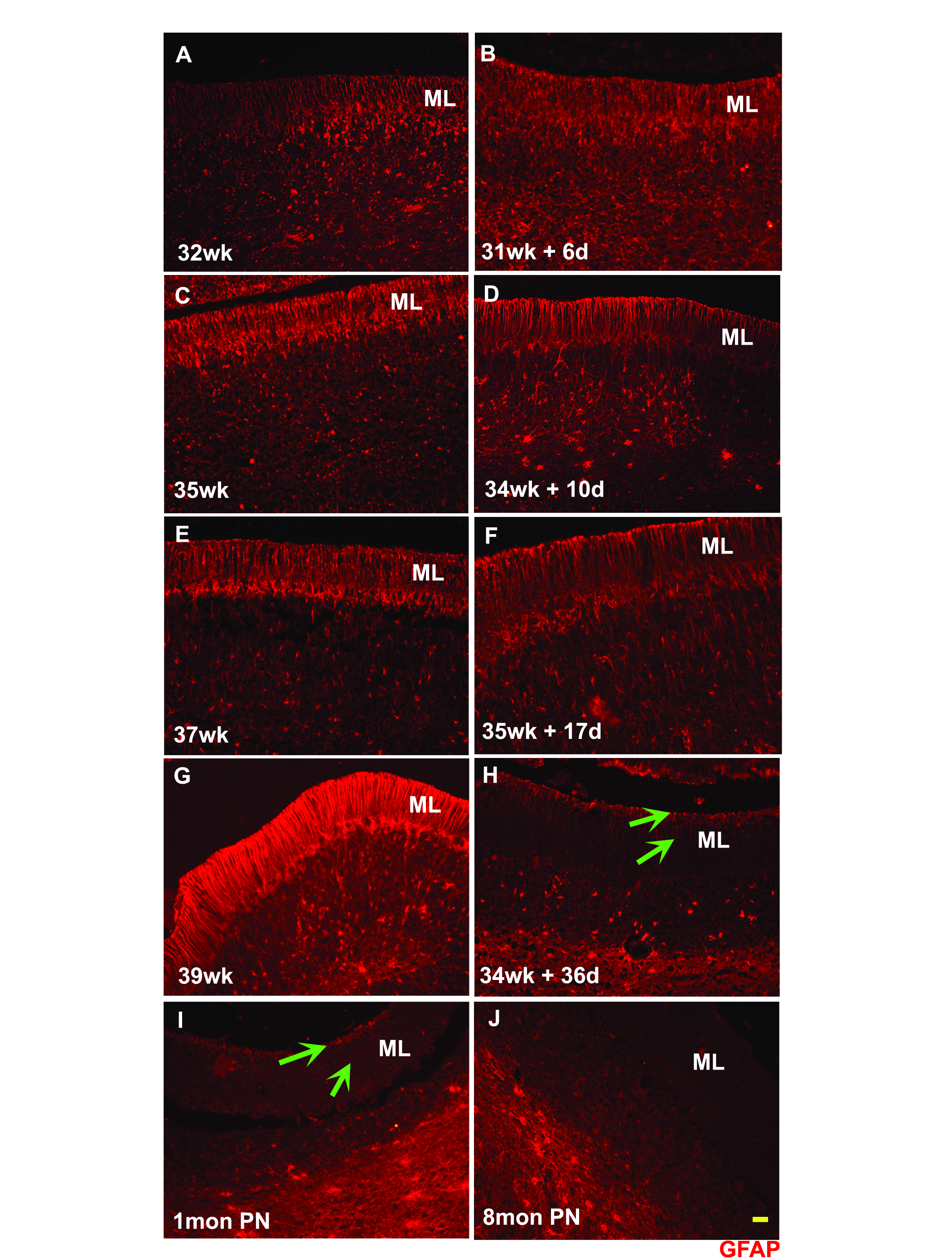

Supplement: Figure S3 — GFAP positive bergmann glia fibres in the ML of the human cerebellum. (A–J) The sections have been stained using GFAP antibody raised in rabbit (red). Scale bar = 50 µm. Abbreviations used - wk = number of gestational weeks, d = number of postnatal days, mon PN = postnatal months- born at term, ML = molecular layer. (TIF) [file pone.0023449.s003.tif]

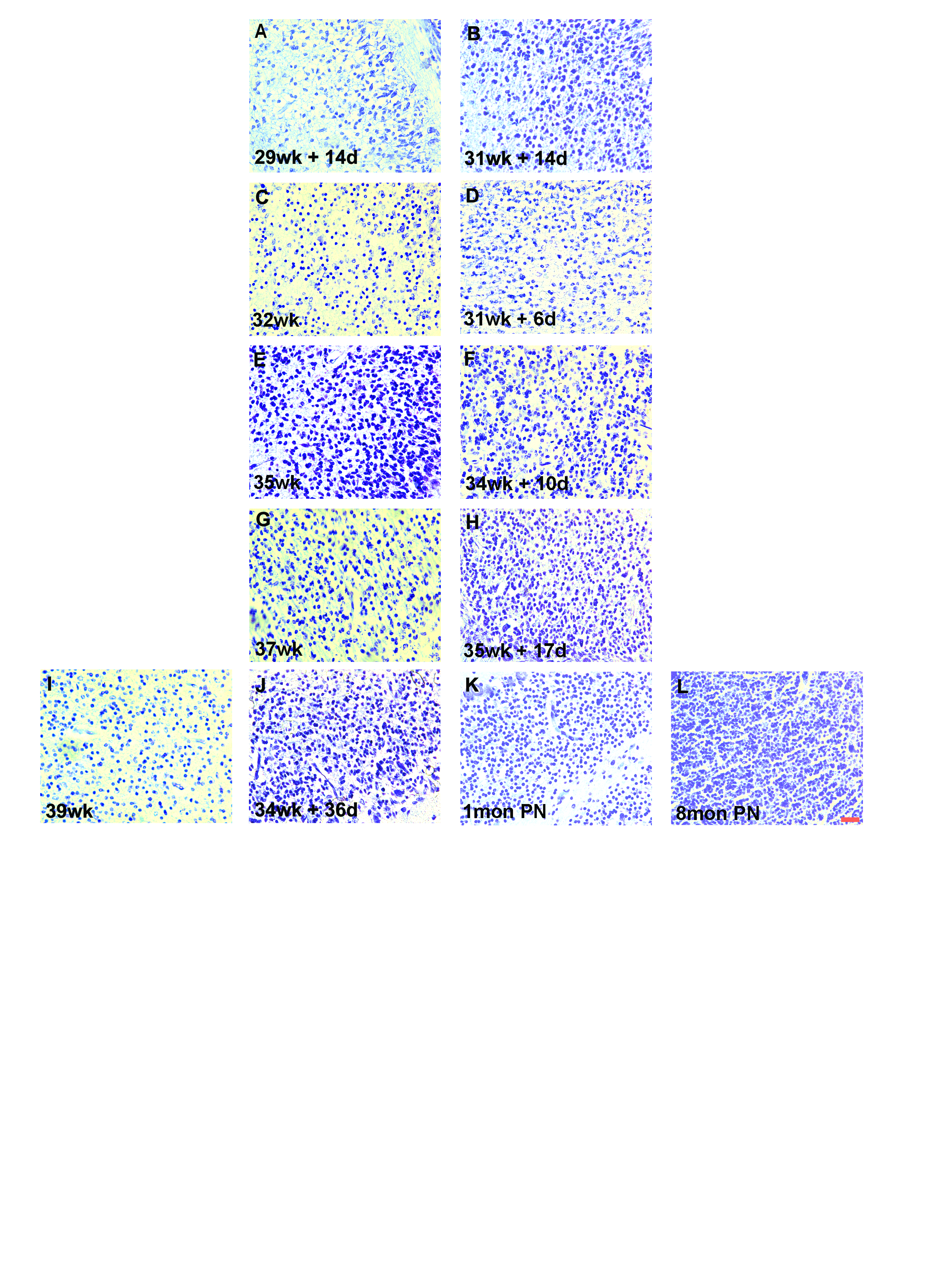

Supplement: Figure S4 — Cell density in the internal granular layer (IGL) increases in samples surviving ex-utero. Abbreviations used - wk = number of gestational weeks, d = number of postnatal days, mon PN = postnatal months- born at term. The sections (A–L) have been stained using Cresyl violet. Scale bar = 20 µm. (TIF) [file pone.0023449.s004.tif]

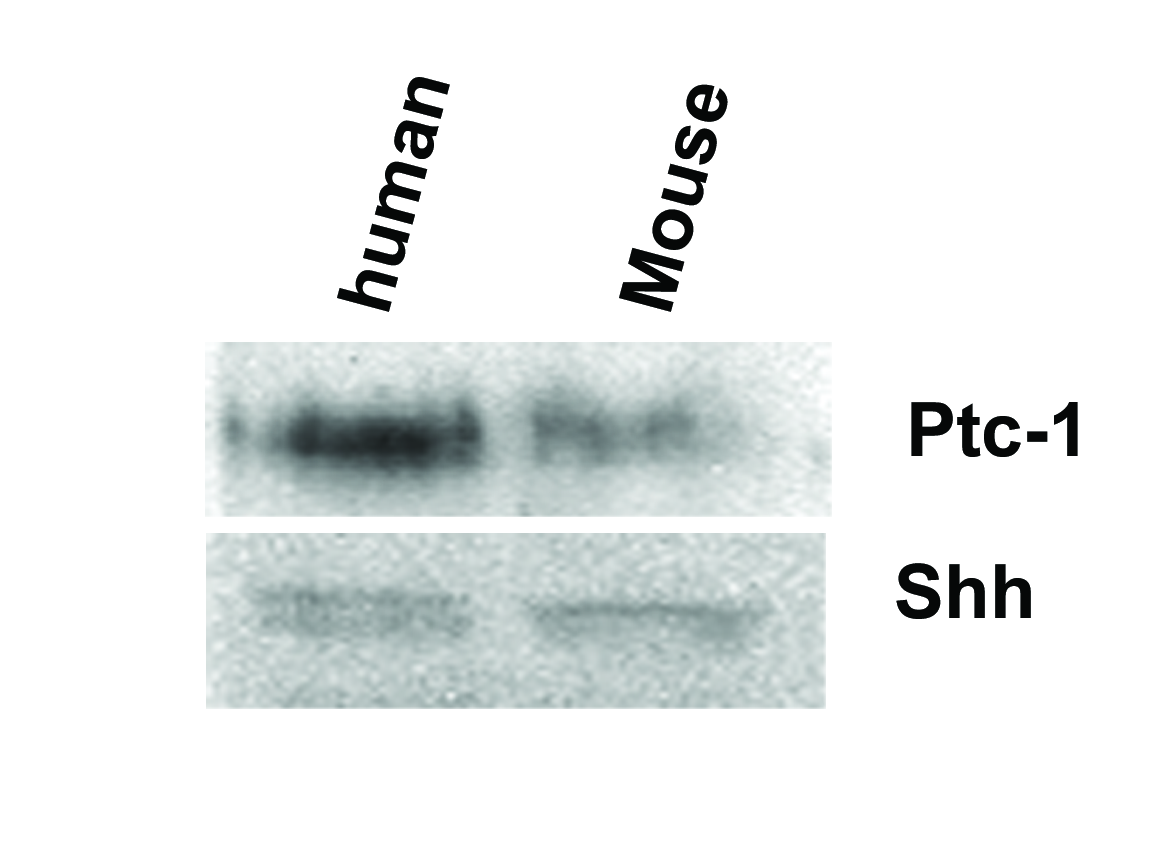

Supplement: Figure S5 — Western blot done for sonic hedgehog, and patched, on human and mouse tissue. (TIF) [file pone.0023449.s005.tif]
