# Supplementary material for: Preterm Delivery Disrupts the Developmental Program of the Cerebellum
Source: PLoS One. 2011 Aug 17;6(8):e23449. doi: 10.1371/journal.pone.0023449 (PMC3157376; doi:10.1371/journal.pone.0023449)
Supplement: Table S1 — List of all the cases used in the current study. The stillborn cases (0 day ex -utero survival) are indicated in red; premature infants (5–36 days survival ex-utero) are indicated in green; postnatal months born at term (1,4 and 8 postnatal months) indicated in blue. Abbreviations used - SVD – spontaneous vaginal delivery; Em LSCS – Emergency Lower segment caesarean section; B and M – bag and mask; NICU – Neonatal Intensive Care Unit; CCF – Congestive Cardiac Failure; Non reactive NST – Non Reactive Non stress test; PROM – Premature rupture of membranes; Vent – Ventilator, VD – vaginal delivery; IPPV – Intermittent Positive Pressure Ventilation; ET – exchange transfusion; Rh – rhesus isoimmunisation; SIMV- Synchronised Intermittent Mandatory Ventilation; CPAP – Continuous positive airway pressure; BT – blood transfusion; PIH – Pregnancy induced hypertension; TPN – Total Parenteral Nutrition; ANA - anti nuclear antibody; IUGR – Intra Uterine Growth Retardation; GCMF – gross congenital mal formation; IUD – Intra Uterine Death; NA - Not apparent, PM - Post mortem, Cause of death 1a/b/c have been mentioned as per international classification of diseases (ICD) codes. Cause of death 2 indicates other pathologies that did not directly contribute to death. (-) in Apgar score indicates non availability of data in the particular case. (DOC) [file pone.0023449.s006.doc]

**Table S1** - List of cases used in the study

| **Gestat-ional age in**  **weeks** | **Post-**  **natal survi-val (in days)** | **Mode of delivery** | **Cause of**  **Preterm**  **delivery** | **APGAR**  **1min** | **APGAR**  **5 min** | **Clinical course** | **Cause of death 1a** | **Cause of death 1b** | **Cause of death 1c** | **Cause of death 2** | **Brain weight in g** | **Any other findings** |
| --- | --- | --- | --- | --- | --- | --- | --- | --- | --- | --- | --- | --- |
| 28 | 0 | SVD | Preterm labour due to  twin pregnancy | 4 | 5 | B&M , NICU, Ventilator | CCF | Anaemia | Twin transfusion synd. |  | 155 g |  |
| 27 | 7 | Em LSCS | Non reactive NST , PROM | 5 | 6 | NICU, Vent, | Pulmonary haemorrhage | Sepsis | Pre  maturity |  | 145 g | Blood culture Enterococcus sp. |
| 29 | 11 | Em LSCS | Maternal eclampsia | 6 | 7 | B&M, Phototherapy | Sepsis | Pre-  maturity |  |  | 158 g | PM blood culture Entero-  haemorrhagic Ecoli |
| 29 | 14 | Assisted Breech VD | N.A. | 4 | 6 | NICU, IPPV, intrauterine  transfusions | Disseminated intravascular coagulopathy | Multi-  organ failure | Non-  immune hydrops fetalis | Hypo-  plastic right kidney | 200g | HPLC - normal, karyotype - normal, Parvovirus B 19 - negative, fetal ECHO - normal |
| 31 | 14 | Em LSCS | Fetal bradycardia | 0 | 4 | B&M , Vent, ET  ( 3 x) | Sepsis due to multiple interventions, CCF | Immune hydrops ( RH ) |  |  | 275 g | High indirect coombs test titres |
| 30 | 20 | Em LSCS | Fetal bradycardia | 5 | 6 | NICU, SIMV, CPAP, BT | germinal matrix hemorrhage with intraventricular extension | Pre-  maturity | Maternal PIH |  | 150 g |  |
| 32 | 0 | Em LSCS | Obstructed labour, cord/hand prolapse | - | - | - | Fetal asphyxia | Obstructed labour | Cord / hand prolapse | Type 1 jejunal atresia | 180 g |  |
| 31 | 5 | Em LSCS | N.A. | - | - | Vent , Pleural tap | CCF | Non immune hydrops fetalis | Placental chor-  Angio-  matosis & intestinal atresia |  | 180 g |  |
| 31 | 6 | SVD | N.A | 6 | 6 | - | Restricted chest expansion | Icthyosis |  |  | 190 g | Family history + |
| 30 | 17 | Em LSCS | Severe PIH , fetal distress | 7 | 7 | NICU, Vent , TPN | Sepsis | Bowel perforation | Pre-  maturity |  | 150 g |  |
| 35 | 0 | LSCS | Fetal bradycardia | 6 | 8 | NICU , Temporary pacing | Cardiac arrest due to malfunctioning pacemaker | Congenital heart block | Neonatal lupus |  | 260 g | ANA ++++  (anti-nuclear antigen staining strong) |
| 34 | 5 | Em LSCS | N. A. | 9 | 9 | NICU, CPAP | Hyaline membrane disease | Pre-  maturity |  | IUGR | 210 g |  |
| 32 | 18 | Em LSCS | PROM | - | - | NICU , vent | Sepsis | Pre-  maturity with PROM |  | Non immune hydrops fetalis | 275 g | PM blood culture : Coagulase negative Staph aureus |
| 34 | 10 | SVD | N.A. | - | - | - | Restricted chest expansion | Harlequins icthyosis | - | - | 180 g | Multiple GCMF : Fish mouth, syndactyly |
| 37 | 0 | SVD | - | - | - | - | Septicemia | Necrotizing enterocolitis | Pre-  maturity | Colonic aganglion-osis | 490 g |  |
| 35 | 17 | Em LSCS | N.A. | 5 | 7 | B&M, NICU, vent. | Pneumonia with septic shock | Acyanotic congenital heart disease , prematurity |  |  | 130 g |  |
| 39 | 0 | Induced VD | IUD | - | - | - | Fetal asphyxia | Cord compression, cord around neck |  |  | 600 g |  |
| 38 | 7 | Forceps ass. VD | PROM | 6 | 7 | NICU, Vent | Lobar pneumonia with sepsis | Prolonged rupture of membrane & meconium aspiration |  | Corpus callosum agenesis  Micro-cephaly, Pachy-gyria | 175 g | Prolonged rupture of membrane. |
| 34 | 36 | Em LSCS | N.A. | 7 | 8 | NICU, Vent | Cardiogenic shock | Aortic coarctation with closed ductus arteriosus |  |  | 380 g |  |
| 40 | 1 mon | SVD | - | - | - | Depressed sensorium since birth , Prog seizures since 2 days of life | Aspiration with pulmonary atelectasis | Refractory seizures | Metabolic etiology |  | 400 g |  |
| 39 | 4 mon | SVD | - | - | - | - | Pulmonary insufficiency | Recurrent pneumonia | Cystic Fibrosis |  | 510 g | SVD |
| 40 | 8 mon | SVD | - | - | - | Bouts of respiratory distress since 2 months of age | Sepsis with acute respiratory distress syndrome | Cirrhosis | Tyrosi- nemia | Cystic dilation of renal tubules | 690 g | Sibling death due to hepatic cirrhosis  (Tyrosinemia), aspergillosis |

**Table S1 -** The table lists all the cases that were used in the current study. The stillborn cases (0 day ex -utero survival) are indicated in red; premature infants (5-36 days survival ex-utero) are indicated in green; postnatal months born at term (1,4 and 8 postnatal months) indicated in blue. Abbrevisations used - SVD – spontaneous vaginal delivery; Em LSCS – Emergency Lower segment caesarean section; B and M – bag and mask; NICU – Neonatal Intensive Care Unit; CCF – Congestive Cardiac Failure; Non reactive NST – Non Reactive Non stress test; PROM – Premature rupture of membranes; Vent – Ventilator, VD – vaginal delivery; IPPV – Intermittent Positive Pressure Ventilation; ET – exchange transfusion; Rh – rhesus isoimmunisation; SIMV- Synchronised Intermittent Mandatory Ventilation; CPAP – Continuous positive airway pressure; BT – blood transfusion; PIH – Pregnancy induced hypertension; TPN – Total Parenteral Nutrition; ANA - anti nuclear antibody; IUGR – Intra Uterine Growth Retardation; GCMF – gross congenital mal formation; IUD – Intra Uterine Death; NA - Not apparent, PM - Post mortem, Cause of death 1a/b/c have been mentioned as per international classification of diseases (ICD) codes. Cause of death 2 indicates other pathologies that did not directly contribute to death. (-) in Apgar score indicates non availability of data in the particular case.
