# Supplementary material for: Preterm Delivery Disrupts the Developmental Program of the Cerebellum
Source: PLoS One. 2011 Aug 17;6(8):e23449. doi: 10.1371/journal.pone.0023449 (PMC3157376; doi:10.1371/journal.pone.0023449)
Supplement: Table S2 — Intensities of staining of sonic hedgehog, calbindin (internal control), patched, smoothened, Gli-1 and Gli-2 in stillborn and preterm infants. A double blind approach has been used for the same. A 0–3 intensity scale to describe the intensity of staining with 0 indicating no staining while 3 indicated highly intense staining. (DOC) [file pone.0023449.s007.doc]

**Table S2 - The intensities of staining of sonic hedgehog, calbindin (internal control), patched, s**moothened, Gli-1 and Gli-2 in stillborn and preterm infants.

| **Age** | **Shh** | **Calbindin** | **Ptc** | **Smo** | **Gli-1** | **Gli-2** |
| --- | --- | --- | --- | --- | --- | --- |
|  |  |  |  |  |  |  |
| 28 wk | 3 | 3 | 3 | 3 | 3 | 3 |
| 27 wk +7d | 3 | 3 | 3 | 3 | 3 | 3 |
| 29 wk + 11d | 3 | 3 | 3 | 3 | 3 | 3 |
| 29 wk + 14d | 3 | 3 | 3 | 3 | 3 | 3 |
| 31 wk + 14d | 3 | 3 | 3 | 3 | 3 | 3 |
| 30 wk +20d | 3 | 3 | 3 | 3 | 3 | 3 |
| 32 wk | 3 | 3 | 3 | 3 | 3 | 3 |
| 31 wk + 5d | 3 | 3 | 3 | 3 | 3 | 3 |
| 31 wk + 6d | 3 | 3 | 3 | 3 | 3 | 3 |
| 30 wk + 17d | 3 | 3 | 3 | 3 | 3 | 3 |
| 35 wk | 3 | 3 | 3 | 3 | 3 | 3 |
| 34 wk + 5d | 3 | 3 | 3 | 3 | 3 | 3 |
| 32 wk + 18d | 3 | 3 | 3 | 3 | 3 | 3 |
| 34 wk + 10d | 2 | 3 | 2 | 2 | 2 | 2 |
| 37 wk | 3 | 3 | 3 | 3 | 3 | 3 |
| 35 wk + 17d | 1 | 3 | 1 | 1 | 1 | 1 |
| 39 wk | 3 | 3 | 3 | 3 | 3 | 3 |
| 38 wk +7d | 2 | 3 | 2 | 2 | 2 | 2 |
| 34 wk + 36d | 1 | 3 | 1 | 1 | 1 | 1 |
| 1 mon | 1 | 3 | 1 | 1 | 1 | 1 |
| 4 mon | 0 | 3 | 0 | 0 | 0 | 0 |
| 8 mon | 0 | 3 | 0 | 0 | 0 | 0 |

3- High intensity of staining

2 - Medium intensity of staining

1 - Low intensity of staining

0 - No staining
