# Supplementary material for: Preterm Delivery Disrupts the Developmental Program of the Cerebellum
Source: PLoS One. 2011 Aug 17;6(8):e23449. doi: 10.1371/journal.pone.0023449 (PMC3157376; doi:10.1371/journal.pone.0023449)
Supplement: Table S3 — The table lists all EGL and ML thickness values that were obtained from stillborn infants at various stage of maturity in comparison to the values from previously published studies. (DOC) [file pone.0023449.s008.doc]

**Table S3 - List of all EGL and ML thickness** values that were obtained from stillborn infants at various stage of maturity in comparison to the values from previously published studies

| Age | EGL thickness in um (Rakic et al, 1971) | EGL thickness in um  (Abraham et al, 2001) | EGL thickness (stillborns)  in um (Data, Haldipur et al) |
| --- | --- | --- | --- |
| 19-20 weeks | 30-35um |  |  |
| 20-21 | 35-40 um |  |  |
| 22-25 | 50-70 um |  |  |
| 27-28 | 50-70 um | 30-45 | 39-44 um |
| 30-32 | 50-70 um | 30-45 | 34-40 um |
| 32-36 | 50-70 um | 40-50 | 34-40 um |
| 36-birth | 50-70 um | 30-40 | 35-45 um |

| 1-6 months | 25-30 um | 20-40 | 0-26 um (1-8 mon) |
| --- | --- | --- | --- |
| 6 months onwards | Disappears by 1^st^ year | 0-10 |

| Age | ML thickness in um (Rakic et al, 1971) | ML thickness in um  (Abraham et al, 2000) | ML thickness  (stillborns)in um  (Data, Haldipur et al) |
| --- | --- | --- | --- |
| 19-20 weeks | 35-40 um |  |  |
| 20-21 | 35-40 um |  |  |
| 22-25 | 40-50 um |  |  |
| 27-28 | 40-50 um | 35-45 | 40-45 um |
| 30-32 | 40-50 um | 35-45 | 37-42 um |
| 32-36 | 70-90 um | 40-50 | 40-70 um |
| 36-birth | 90-110 um | 40-60 | 55-70 um |

| 1-6 months | 200 um | 70-240 | 90-280 um (1-8 mon) |
| --- | --- | --- | --- |
| 6 months onwards | 300 um | 150-220 |
