# Supplementary material for: Preterm Delivery Disrupts the Developmental Program of the Cerebellum
Source: PLoS One. 2011 Aug 17;6(8):e23449. doi: 10.1371/journal.pone.0023449 (PMC3157376; doi:10.1371/journal.pone.0023449)
Supplement: Table S4 — List of all the cases that were used in the current study that have been classified according to their cause of death. (DOC) [file pone.0023449.s009.doc]

Table S4 –List of cases classified according to the cause of death

| Cause of Death | No. of still borns | Age | No of preterms | Age |
| --- | --- | --- | --- | --- |
| Anemia | 1 | 28 wk | 0 | 0 |
| Congestive cardiac failure | 2 | 28 wk, 32 wk | 1 | 31 wk + 14d |
| Cardiac arrest | 1 | 35 wk | 0 | 0 |
| Congenital heart block | 1 | 35 wk | 0 | 0 |
| Cardiogenic shock | 0 | 0 | 1 | 34wk + 36d |
| Colonic agangliosis | 1 | 37 wk | 0 | 0 |
| Congenital heart disease | 0 | 0 | 1 | 35 wk + 17d |
| Cord compression around neck | 1 | 39 wk | 0 | 0 |
| Ductus arteriosis | 0 | 0 | 1 | 34 wk +36d |
| Disseminated intravascular coagulopathy | 0 | 0 | 1 | 29 wk +14d |
| Fetal asphyxia | 2 | 32 wk, 39 wk | 0 | 0 |
| Germinal matrix hemorrhage | 0 | 0 | 1 | 30 wk +20d |
| Hydrops fetalis | 0 | 0 | 3 | 29 wk +14d , 31wk +5d, 32wk+18d |
| Hypoplastic kidney |  | 0 | 1 | 29wk+14d |
| Hyaline membrane disruption | 0 | 0 | 1 | 34 wk+5d |
| Immune hydrops | 0 | 0 | 1 | 31wk+14d |
| Intra uterine growth retardation | 0 | 0 | 1 | 34wk+5d |
| Ichthyosis | 0 | 0 | 2 | 31wk+6d, 34wk+10d |
| Jejunal atresia | 1 | 32 wk | 1 | 31wk+5d |
| Maternal PIH | 0 | 0 | 1 | 30wk+20d |
| Multivascular failure | 0 | 0 | 1 | 29wk+14d |
| Neonatal lupus | 1 | 35 wk | 0 | 0 |
| Necrotizing enterocolitis | 1 | 37 wk | 0 | 0 |
| Obstructed labor | 1 | 32 wk | 0 | 0 |
| Pulmonary hemorrhage |  |  | 1 | 27wk+7d |
| Prematurity | 1 | 37 wk | 7 | 27wk+7d, 29wk+11d, 31wk+14d, 30wk+17d, 32wk+18d, 35wk+17d |
| Pneumonia | 0 | 0 | 2 | 35wk+17d, 38wk+7d |
| Restricted chest expansion | 0 | 0 | 2 | 31wk+6d, 34wk+10d |
| Sepsis | 0 | 0 | 6 | 27wk+7d, 29wk+11d, 31wk+14d, 30wk+17d, 32wk+18d, 38wk+7d |
| Twin transfusion syndrome | 1 | 28 wk | 0 | 0 |
