# Supplementary material for: Preterm Delivery Disrupts the Developmental Program of the Cerebellum
Source: PLoS One. 2011 Aug 17;6(8):e23449. doi: 10.1371/journal.pone.0023449 (PMC3157376; doi:10.1371/journal.pone.0023449)
Supplement: Table S5 — List of all primary and secondary antibodies used in the study, including their type, dilution and company name. (DOC) [file pone.0023449.s010.doc]

Table S5 – List of antibodies used in the study

| Name of the antibody | Type | Dilution used | Company |
| --- | --- | --- | --- |
| PCNA | Mouse monoclonal | 1:400 | Cell signalling Technology |
| Double cortin | Rabbit polyclonal | 1:150 | Abcam |
| Gli-1 | Rabbit polyclonal | 1:250 | Abcam |
| Gli-2 | Rabbit polyclonal | 1:250 | Abcam |
| Patched | Rabbit polyclonal | 1:200 | Abcam |
| Smoothened | Rabbit polyclonal | 1:200 | Abcam |
| ITPR | Rabbit polyclonal | 1:200 | Abcam |
| GFAP | Rabbit polyclonal | 1:3000 | DAKO |
| Sonic Hedgehog | Rabbit polyclonal | 1:100 | Santa Cruz |
| Beta-III-Tubulin | Mouse monoclonal | 1:3000 | Promega |
| Calbindin | Rabbit polyclonal | 1:2000 | Swant |
| Biotinylated secondary | Rabbit | 1:250 | Vector laboratories |
| Alexa fluor 488 | Mouse | 1:1000 | Molecular probes |
| Alexa fluor 594 | Mouse | 1:1000 | Molecular probes |
| Alexa fluor 488 | Rabbit | 1:1000 | Molecular probes |
| Alexa fluor 594 | Rabbit | 1:1000 | Molecular probes |
